# PET/CT reading for relapse in non-small cell lung cancer after chemoradiotherapy in the PET-Plan trial cohort

**DOI:** 10.1186/s40644-023-00567-6

**Published:** 2023-05-17

**Authors:** Alexander Brose, Kerstin Michalski, Juri Ruf, Marco Tosch, Susanne M. Eschmann, Mathias Schreckenberger, Jochem König, Ursula Nestle, Matthias Miederer

**Affiliations:** 1 Department of Translational Imaging in Oncology, National Center for Tumor Diseases (NCT/UCC) Dresden, Germany: German Cancer Research Center (DKFZ), Heidelberg, Faculty of Medicine and University Hospital Carl Gustav Carus, University of Technology Dresden (TUD), Helmholtz-Zentrum Dresden-Rossendorf (HZDR), Dresden, Germany; 2 Department of Diagnostic and Interventional Radiology, University Hospital Giessen, Giessen, Germany; 3 Department of Nuclear Medicine, University Hospital Würzburg, Würzburg, Germany; 4 Department of Nuclear Medicine, Medical Center, Faculty of Medicine, University of Freiburg, Freiburg, Germany; 5 Department of Nuclear Medicine, Helios University Hospital Wuppertal, Wuppertal, Germany; 6 Department of Medicine, Faculty of Health, University of Witten/Herdecke, Witten, Germany; 7 Department of Nuclear Medicine, Marienhospital Stuttgart, Stuttgart, Germany; 8 Department of Nuclear Medicine, University Medical Center Mainz, Johannes Gutenberg-University Mainz, Mainz, Germany; 9 Institute of Medical Biostatistics, Epidemiology and Informatics (IMBEI), University Medical Center Mainz, Johannes Gutenberg-University Mainz, Mainz, Germany; 10 Department of Radiation Oncology, Kliniken Maria Hilf, Mönchengladbach, Germany; 11 Department of Radiation Oncology, University Hospital Freiburg, Freiburg, Germany

**Keywords:** PET/CT, NSCLC, Oncology, Tumor recurrence, Relapse criteria

## Abstract

**Background:**

Current studies indicate that fluorine-18-fluorodeoxyglucose positron emission tomography/ computed tomography ([^18^F]FDG PET/CT) is the most accurate imaging modality for the detection of relapsed locally advanced non-small cell lung cancer (NSCLC) after curatively intended chemoradiotherapy. To this day, there is no objective and reproducible definition for the diagnosis of disease recurrence in PET/CT, the reading of which is relevantly influenced by post radiation inflammatory processes. The aim of this study was to evaluate and compare visual and threshold-based semi-automated evaluation criteria for the assessment of suspected tumor recurrence in a well-defined study population investigated during the randomized clinical PET-Plan trial.

**Methods:**

This retrospective analysis comprises 114 PET/CT data sets of 82 patients from the PET-Plan multi-center study cohort who underwent [^18^F]FDG PET/CT imaging at different timepoints for relapse, as suspected by CT. Scans were first analyzed visually by four blinded readers using a binary scoring system for each possible localization and the associated reader certainty of the evaluation. Visual evaluations were conducted repeatedly without and with additional knowledge of the initial staging PET and radiotherapy delineation volumes. In a second step, uptake was measured quantitatively using maximum standardized uptake value (SUVmax), peak standardized uptake value corrected for lean body mass (SULpeak), and a liver threshold-based quantitative assessment model. Resulting sensitivity and specificity for relapse detection were compared to the findings in the visual assessment. The gold standard of recurrence was independently defined by prospective study routine including external reviewers using CT, PET, biopsies and clinical course of the disease.

**Results:**

Overall interobserver agreement (IOA) of the visual assessment was moderate with a high difference between secure (ĸ = 0.66) and insecure (ĸ = 0.24) evaluations. Additional knowledge of the initial staging PET and radiotherapy delineation volumes improved the sensitivity (0.85 *vs* 0.92) but did not show significant impact on the specificity (0.86 *vs* 0.89). PET parameters SUVmax and SULpeak showed lower accuracy compared to the visual assessment, whereas threshold-based reading showed similar sensitivity (0.86) and higher specificity (0.97).

**Conclusion:**

Visual assessment especially if associated with high reader certainty shows very high interobserver agreement and high accuracy that can be further increased by baseline PET/CT information. The implementation of a patient individual liver threshold value definition, similar to the threshold definition in PERCIST, offers a more standardized method matching the accuracy of experienced readers albeit not providing further improvement of accuracy.

## Background

In the western world, lung cancer is the second most common malignant tumor entity in both sexes and the most common cause of cancer-related deaths, being accountable for one fourth of all cancer deaths [1, 2]. Approximately two-thirds of patients are deemed inoperable by the time of initial diagnosis due to locally advanced tumor stage or metastatic disease [3]. For those patients with locally advanced stage II or III non-small cell lung cancer (NSCLC) who are not suitable for surgery, chemoradiotherapy represents a curative therapy approach.

At present time, fluorine-18-fluorodeoxyglucose positron emission tomography/ computed tomography ([^18^F]FDG PET/CT) represents the most accurate imaging modality for staging, planning of radiation therapy, treatment response evaluation and relapse diagnosis for NSCLC [4–6]. The multinational prospective randomized clinical trial PET-Plan (ARO-2009–09) has shown PET/CT is even suitable for imaging-based reduction of radiation therapy target volumes and could lead to an improved loco-regional tumor control [7]. Although well-established criteria for the definition of treatment response exist [8], few studies have focused on objective criteria for the evaluation of locoregional and distant disease recurrence [9]. PET/CT is an integral part of the diagnostic workup to guide locoregional treatment for both initial diagnosis and for suspected locoregional relapse in NSCLC. However, due to inflammatory changes that are particular common in lungs that are exposed to stress due to radiation and chronic inflammation PET reading is challenging [10, 11]. These changes are summarized as Radiation Induced Lung Disease (RILD) and encompass an early phase radiation pneumonitis and late phase radiation fibrosis. Findings of RILD typically involve a diffuse FDG-uptake in an area of lung tissue with septal thickening and ground glass opacities within weeks to months after radiation therapy. A secure visual evaluation with substantial interobserver agreement (IOA) is feasible in these cases, but only if a meticulous visual response assessment technique is used [12, 13]. Structured harmonization processes have shown to improve quality of visual PET/CT evaluation [14]. Uptake quantification in PET using parameters like the maximum standardized uptake volume (SUVmax) have long been hoped to achieve similar certainties compared to visual assessment. In this aspect, standardized criteria for e.g. response assessment have been developed, such as the PET Response Criteria in Solid Tumor (PERCIST) which put emphasis on the therapy response compared to the baseline state or previous assessments [15]. In a more clinical setting in which the experience of the reader often varies, objectifiable criteria showed the potential to improve and augment the visual reader assessment [16]. The aim of this study was to assess different approaches for relapse evaluation and to compare the accuracy of a quantitative and patient individual threshold-based model to the visual assessment of experienced physicians.For the analysis presented here, PET/CT scans that had been obtained during the PET-Plan trial were reassessed and compared to the quality assured primary and secondary endpoints of the trial.

## Materials and methods

### Patient population and reference standard

In the prospective international PET-Plan trial (ARO-2009–09), patients with locally advanced NSCLC underwent FDG-PET-based simultaneous chemoradiotherapy [7]. Follow up was done by three-monthly CT scans. In case of suspected recurrence an FDG-PET/CT was mandatory by protocol. The primary endpoint of the trial was loco-regional recurrence free survival, so loco-regional tumor control was quality assured with the support of an expert panel reviewing follow up imaging and having full access to the medical charts 6 month after end of recruitment of all study patients. Loco-regional recurrence as gold standard of the trial and in the present analysis was defined by progression in imaging and/or biopsy and documented separately for primary tumor, mediastinal lymph node stations and distant metastases [14]. From a total of 205 patients of the PET-Plan trial cohort those who have had CT-suspected relapse at any timepoint after curative-intent chemoradiotherapy and consecutively underwent [^18^F]FDG PET/CT imaging were identified. The available image timepoints were identified from the trial database and the respective image datasets were retrospectively collected from the participating centers. Patients were only included if they had received at least one protocol mandated FDG PET/CT-scan in the follow-up period. A total of 114 PET/CT image datasets of 82 patients from five centers were available and could be included in this retrospective analysis. PET/CT image datasets were re-pseudonymized. Data from the PET Plan study trial database, including the documentation about the quality assured primary and secondary endpoints, was available for the documented outcome of the patients. If one of the study endpoints was reached, further imaging of the patient was excluded from this analysis. The present analysis was approved by the competent ethics committee in addition to the main trial (Mainz, protocol no. 10011/18).

### Imaging protocol

All patients had undergone PET/CT imaging within 3 weeks prior to radiotherapy and by the time of suspected tumor recurrence. Whole-body PET scans had been acquired using three different models of PET/CT scanners (Siemens Biograph 16, Philips Gemini GXL, Philips Gemini TF16) with full-dose contrast-enhanced or low-dose non-contrast-enhanced CT for attenuation correction and anatomical co-registration. Image acquisition followed the mandatory PET-Plan trial protocol [7].

### Image analysis

Visual assessment was performed by 4 experienced physicians in the field of nuclear medicine (*KM*, *JR*, *MT*, *MM*) and followed a standardized protocol with a binary scoring system for lung (yes/ no), mediastinal (yes/ no) and distant recurrence (yes/ no), followed by statements about the certainty (secure/ insecure). FDG-positive lymph nodes were allocated using an anatomical atlas [17] and the definition for FGD-positivity followed the general protocol criteria of the PET-Plan trial [14]. Each observer was asked to first evaluate the PET/CT images by the time of suspected relapse for possible lung, mediastinal and distant recurrence separately, to conclude if there was disease relapse in general, and to state whether or not the reader felt certain with the assessment. The observers then received additive information about the individual patients’ treatment, which included the initial staging PET and the dedicated radiotherapy delineation volumes. They were then asked to repeat the initial evaluation of possible tumor recurrent sites and to again state whether or not they felt certain with the assessment.

Quantitative analyses were performed by a trained radiology resident (*AB*) using a commercial software (Hybrid 3D Tumor Finder V2.2, Hermes Medical Solutions, Stockholm, Sweden). The PET parameters, maximum standardized uptake value (SUVmax) and peak standardized uptake value corrected for lean body mass (SULpeak), and lean body mass were defined as described in the updated PET Response Criteria in Solid Tumor [15]. The liver threshold-based individual relapse evaluation was performed following the threshold definition in PET Residual Disease in Solid Tumor criteria using a region of interest (ROI) with a diameter of 3 cm in the right liver lobe [18]. Tumor recurrence was then defined as a semi-automated ROI showing post-therapy PET FDG uptake higher than 1.5 × SULmean + 2 × SD of the liver ROI in analogy to the PERCIST- and PREDIST criteria [18, 19].

### Statistical analysis

Statistical analyses were performed using SPSS v27 (IBM, Armonk, NY). For the comparison of the liver threshold-based assessment to the visual assessment, an individual observer consensus was built by majority rule. In case of a draw, a fifth observer determined the outcome for the individual image dataset. Interobserver agreement (IOA) and intercriteria agreement (ICA) were calculated using Cohens ĸ and interpreted as described by Landis and Koch [20]. IOA and ICA were calculated lesion-based (lung primary, mediastinum, distant recurrence), and patient-based (disease relapse = locoregional and/or distant recurrence). For statistical comparison between groups paired t-test or McNemar test were performed when applicable. Sensitivity, specificity, coefficient of variation and correlation coefficient were calculated examination-based using SPSS. All *p*-values reported are two-sided. A *p*-value less than 0.05 was considered statistically significant.

## Results

### Patients characteristics

Eighty-two patients with CT-suspected relapse after chemoradiotherapy of locally advanced NSCLC were included in this analysis, with a total of 114 consecutive PET/CT-scans. For 78 of them, a final judgement of either loco-regional progression (*n* = 22) or distant progression (*n* = 28) or both (*n* = 28) was documented in the study database. 17 patients showed disease relapse (loco-regional and/or distant recurrence) within the first six months after treatment. 64 patients underwent PET/CT once during follow-up, 18 patients received more than one PET/CT. Patient characteristics are shown in Table 1. The mean time to CT-suspected relapse ± standard deviation was 18 ± 16 months. 21 out of 114 PET/CT examinations were performed within the first six months after chemoradiotherapy. 76 out of 114 PET/CT examinations were classified as relapse by the observer consensus of the visual assessment, whereas the liver threshold analysis classified 68 out of 114 examinations as relapse. The mean SUL in the liver was 1.68 ± 0.46 and 1.83 ± 0.55 (within-patient coefficient of variation, 20.9% and intraclass correlation coefficient, 0.68) on baseline study and follow up, respectively.

**Table 1:**
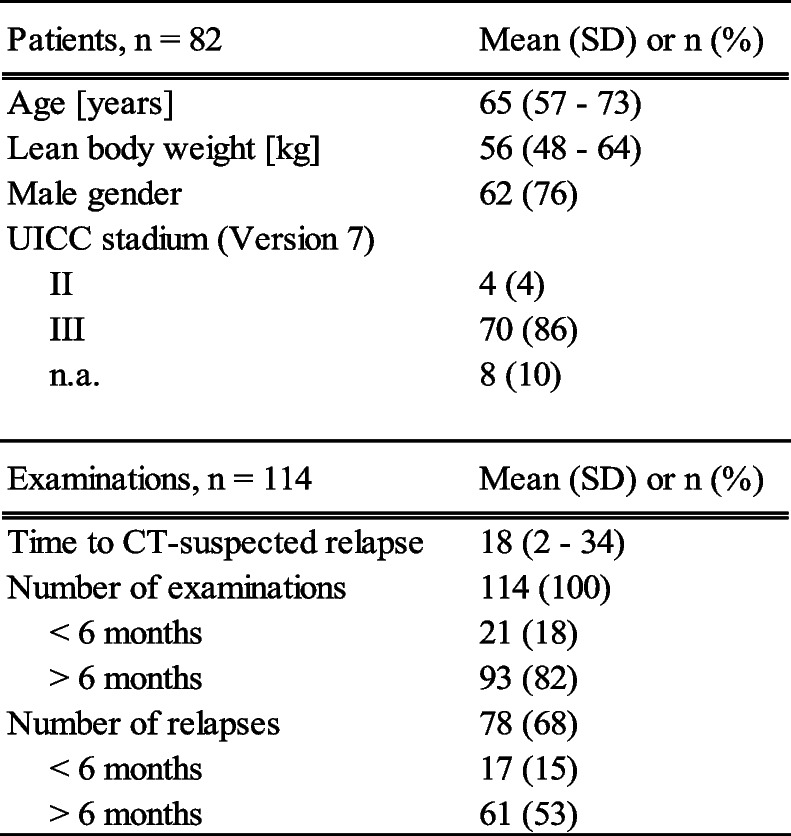
Patient characteristics

*LBW* lean body weight, *UICC* Union for International Cancer Control, *n.a.* not available.

### Visual analysis

We report interobserver agreement (IOA) between 4 observers on 114 examinations. Overall IOA for the examination based evaluation of disease relapse was moderate (ĸ = 0.57) with substantial agreement between the observers for the assessment of tumor recurrence in the primary lung tumor (ĸ = 0.61) and distant recurrence (ĸ = 0.69). The evaluation of lymph node recurrence in the mediastinum showed moderate interobserver agreement (ĸ = 0.58). Comparing the evaluation without and with additional knowledge about the initial staging PET and the dedicated radiotherapy delineation volumes, there was no statistically significant difference in kappa values of IOA. Additionally, we classified examinations as unequivocal if all 4 observers reported to be sure in their judgement about the examined entity, and equivocal otherwise. In secure cases where all observers felt certain in their assessment, IOA showed substantial agreement for the assessment of the primary lung tumor (ĸ = 0.74, *n* = 93), the mediastinum (ĸ = 0.65, *n* = 102) and possible disease relapse in general (ĸ = 0.66, *n* = 106). In equivocal cases, with at least one observer stating insecurity in the evaluation, IOA showed fair agreement for the primary lung tumor (ĸ = 0.22, *n* = 21), slight agreement for the mediastinum (ĸ = 0.15, *n* = 12) and fair agreement for disease relapse in total (ĸ = 0.24, *n* = 8). A statistically significant difference could be observed between the groups of unequivocal and equivocal evaluations (*p* < 0.05). The ĸ values for the IOA of the visual assessment can be found in Table 2.

**Table 2:**
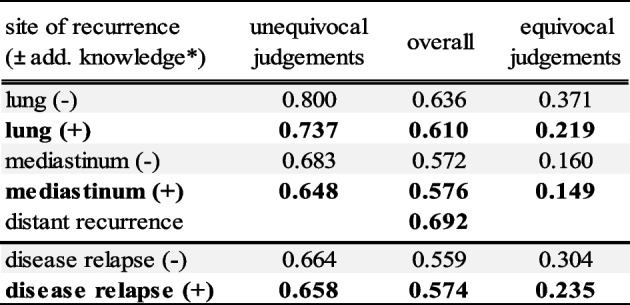
Interobserver agreement of the visual assessment

* (-) additional information about the initial staging PET and the dedicated radiotherapy delineation volumes was not provided, ( +) additional information about the initial staging PET and the dedicated radiotherapy delineation volumes was provided

Pooled sensitivity and specificity of the visual evaluation by the four observers are displayed in Table 3. We found a sensitivity of 74%, 83% and 79% and a specificity of 71%, 85% and 90% in the evaluation of pulmonary, mediastinal and distant recurrence, respectively. The overall evaluation of the whole-body tumor recurrence differed from the single sites, as patients could have tumor recurrence in multiple sites simultaneously. The sensitivity and specificity for the correct diagnosis of disease relapse in total was 92% and 89%, respectively. Additional knowledge of the initial staging PET and radiotherapy delineation volumes improved the sensitivity significantly (85% *vs* 92%) but did not show a statistically significant impact on the specificity (86% *vs* 89%).

**Table 3:**
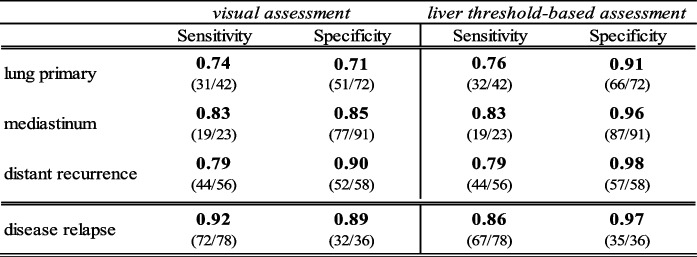
Sensitivity and Specificity of the visual and the threshold-based assessment

### Quantitative analysis

Different fixed threshold values of SUVmax and SULpeak for the definition of tumor relapse were defined and tested for their sensitivity and specificity regarding the correct diagnosis of disease recurrence (Table 4). A fixed SUVmax greater than 3.5 and SULpeak greater than 2.0 showed a good sensitivity of 88%, but poor specificity of 64% and 58%, respectively. Taking in account a higher fixed threshold value for SUVmax greater than 6.0 and SULpeak greater than 3.0 will raise the specificity to 92% and 94% but will reduce the sensitivity of the correct diagnosis to 73%. In contrast, the liver threshold-based individual relapse evaluation showed a sensitivity of 86% and a specificity of 97%, which makes it the most favorable combination of the tested threshold values.

**Table 4:**
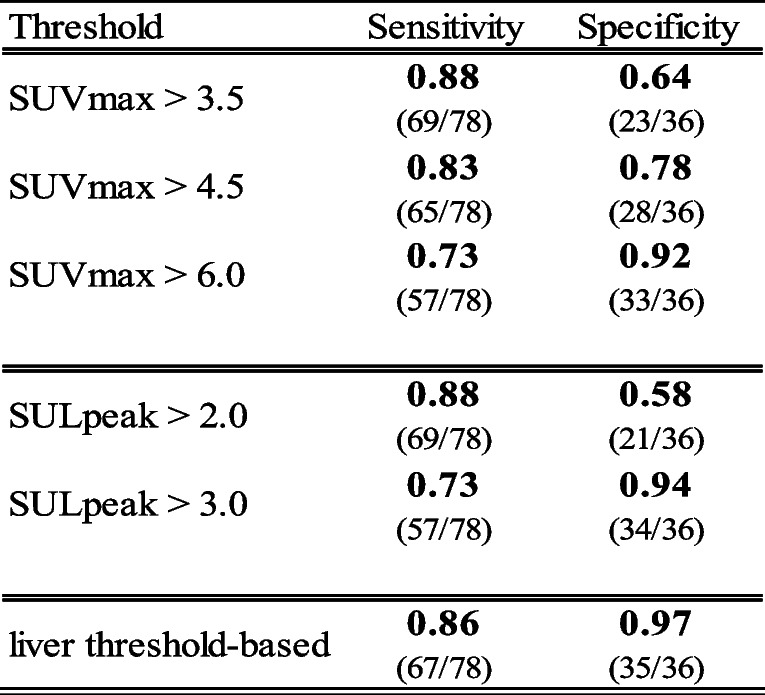
Sensitivity and Specificity of different thresholds

### Time of suspected relapse

The results of the visual and the liver threshold-based evaluation methods were divided into different time intervals of suspected disease relapse. The results within and after 6 months following chemoradiotherapy are shown in Table 5. The observer consensus of the visual assessment shows a significantly higher sensitivity in both time periods with 94% vs 82% and 92% vs 87% when compared to the liver threshold-based evaluation (*p* < 0.05). Especially remarkable is the high observer sensitivity of 100% (6/6) when it comes to evaluate the lung parenchyma within the first six months after therapy. Nevertheless, corresponding specificity for this interval is 33%. Both visual and quantitative evaluation method reach higher specificity rates for the evaluation of the lung and the mediastinum in the interval more than 6 months after chemoradiotherapy. The liver threshold-based evaluation shows an overall higher specificity, with the highest specificity more than 6 months after the end of chemoradiotherapy. The visual evaluation shows comparable results with a specificity of 94% beyond 6 months after therapy. Differences in specificity between the two assessment methods were not statistically significant (*p* > 0.05). Intercriteria agreement was substantial, with moderate agreement in the diagnosis of relapse within the first six months after therapy and almost perfect agreement thereafter (Table 6).

**Table 5:**
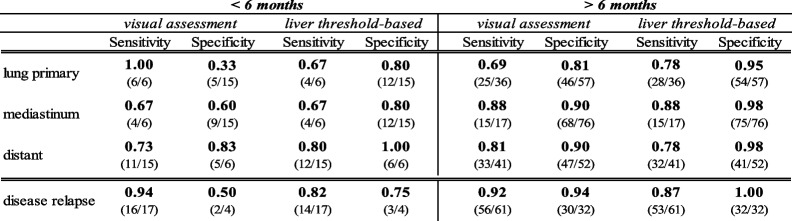
Sensitivity and specificity of the visual and threshold-based assessment at different timepoints of suspected relapse

**Table 6:**
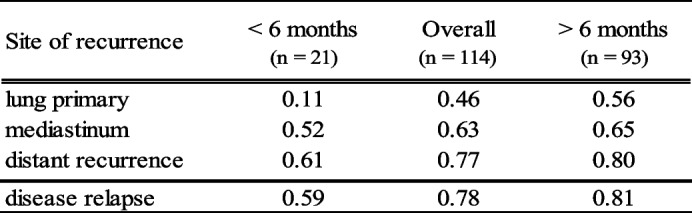
Intercriteria agreement between visual and liver threshold based assessment methods

## Discussion

**Fig. 1:**
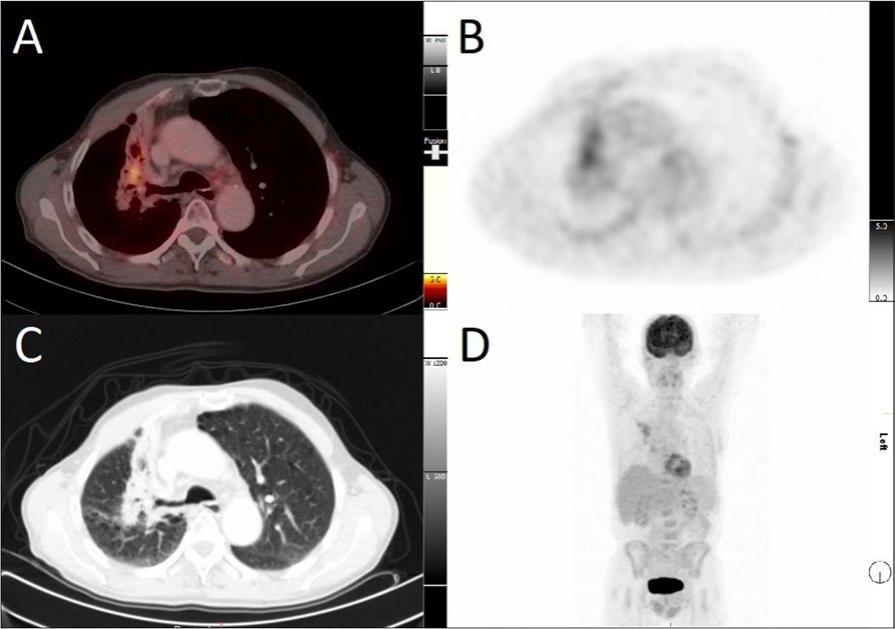
Inflammatory changes in fibrotic tissue after radiation. Axial fused PET/CT image (**A**), attenuation corrected PET image (**B**), CT image in lung window (**C**) and 3D VRT images of the whole body (**D**) showing fibrotic changes without locoregional tumor recurrence. PET shows diffuse FDG uptake (SUVmax 3.5, SULpeak 2.7) of the right upper lobe 5 years after radiation therapy

The superiority of PET/CT over CT for the detection of malignancy in lung nodules, mediastinal staging [4] and detection of unsuspected distant metastases [5], as well as response evaluation and restaging [6] has been demonstrated in various studies in the past two decades. It is often difficult to differentiate between therapy effect and tumor recurrence particularly when tumor, surrounding tissue and healthy lung parenchyma have been treated by chemoradiotherapy (Fig. 1). In these complex situations PET/CT plays a crucial role in guiding therapy and stratifying prognosis [11]. Our study is one of the first to investigate a well-defined cohort from a large multicenter trial and compare PET/CT findings of visual and quantitative assessment methods. The particular strength is the well-validated endpoint of the PET-Plan study and the stratification of relapse according to its site.

General approaches to read PET/CT include visual and quantitative assessment strategies. Visual evaluation is the most common method to interpret FDG-avid findings in daily routine. Appropriate strategies for recurrence detection include definitions where focal FDG uptake above liver uptake likely reflects tumor and diffuse uptake lower than liver signal rather reflects post therapeutic changes such as inflammation or fibrosis. The definition of Peter Mac criteria and Hopkins criteria represent this approach and intend to define reproducible study criteria for solid tumors [21, 22]. Recent studies demonstrate high agreement between observers for a visual assessment with predefined criteria, when conducted by experienced readers [10, 12, 13]. Our study supports those findings, especially within the first six months after radiation therapy when postradiation inflammatory changes, e. g. radiation pneumonitis, hinder the assessment of locoregional tumor recurrence. In this scenario, the visual assessment better distinguishes between tumor recurrence and pneumonitis, when compared to threshold-based assessments. This is most likely because of the better discrimination between focal FDG-uptake in tumor recurrence and diffuse uptake in RILD of the irradiated primary tumor site. Additional information about the initial tumor staging and radiation therapy delineation lead to further improvement in the visual evaluation. We could observe an improvement in reader certainty, sensitivity and specificity in the diagnosis of tumor recurrence when the reader is provided with the relevant baseline information. The information about the primary site and initial FDG-uptake of the tumor and the radiation delineation volumes lead to a more accurate visual assessment. We could also show that a high degree in confidence comes with a high level of agreement between the separate observer assessments. As expected, IOA is worse when reader evaluation is rated as insecure and findings in PET/CT are equivocal. With this knowledge, clinical reports will possibly benefit from a statement about the level of certainty.

With the potential of imaging derived uptake quantification in PET/CT, quantitative parameters have long been hoped to allow fixed definitions of imaging findings. SUVmax as a commonly used parameter in daily routine represents a single voxel with the highest uptake value and is therefore easy to use. However, with the improvement of scanners, smaller voxel sizes and variation of matrix sizes, SUVmax is not preferable. Single voxel values are not as reproducible as larger region of interests (ROI) [23, 24] and caution must be applied when assessing small changes in SUVmax induced by treatment [25]. Due to the additional variation in weight and body fat composition of a patient during cancer therapy some studies even suggest adjusting the SUV to the patient’s lean body mass and could show higher accuracy for therapy monitoring in test–retest-studies [19, 26]. In our study, both SUVmax and SULpeak lead to similar sensitivity and specificity in diagnosing disease relapse, when used as fixed thresholds.

SULpeak which is corrected for lean body mass and describes a ROI rather than a single voxel, is the fundamental parameter in PERCIST [19]. Most studies use these criteria, defined by Wahl et al*. *[19], to graduate therapy assessment in 4 categories. In the entity of locally advanced rectal carcinoma these criteria were simplified by Maffione et al. [18]. Only 2 categories are used to differ between complete metabolic response (CMR) and residual disease [27]. These PREDIST criteria have proven to better distinguish between residual cancer tissue and postactinic inflammation, which is likely because of an implemented individual liver threshold definition [18]. In our study, we integrated the threshold definition of PREDIST and translated it into the two categories of CMR and disease relapse. This showed much better sensitivity and specificity in diagnosing disease relapse than fixed threshold definitions for SUVmax and SULpeak. It also allows for an assessment of PET/CT for an individual timepoint with no need for previous imaging studies for comparison, as in e.g. PERCIST.

Intercriteria agreement (ICA) between our visual and quantitative evaluation was consistently substantial and even showed almost perfect agreement in relapse assessment of more than 6 months after therapy. Differences exist in the evaluation of different timepoints after therapy: rigid threshold-based assessments are more likely to fail in differentiating inflammation and tumor recurrence. This is one possible explanation for our observation of lower ICA within the first 6 months after therapy and higher ICA after 6 months. This interpretation is supported by the very low and only slight agreement between visual and quantitative evaluation of the lung parenchyma within the first 6 months (ĸ = 0.11) when post-radiation inflammation confounds PET/CT reading. Our findings in the early post therapeutic phase support the superiority of the visual assessment in differentiating between radiation pneumonitis and locoregional tumor recurrence. Nevertheless, quantitative parameters are highly reproducible and especially unexperienced readers might benefit from the implementation of quantitative criteria [16]. In our study we could prove the superiority of an individual threshold-based assessment over the mere application of fixed threshold of SUVmax or SULpeak. In this context the concept of liver normalization is especially attractive for comparing tumor activity and when measured in healthy organs even shows better within-patient coefficient of variation than mediastinum in test–retest studies [28]. In PERCIST it serves as threshold definition and quality assurance between scans. Therefore, differences in liver uptake must not exceed a total value of 0.3 SUL units or more than 20% between imaging studies, as defined by Wahl et al. [15]. Normalizing uptake to liver background potentially ensures quality of scans from test to retest, with normal within-patient coefficient of variation ranging from 10 to 15% [19]. In our study we calculated a value of 20.9%, which is probably due to longer intervals between patient examinations as compared to studies in literature and especially because different scanners were used, which should be avoided if possible [29]. However, the calculated intraclass correlation coefficient, as a marker of reproducibility for the individual liver background, was very high with a value of 0.68 and underlines the comparability of the SUVs from the different scanners used in this study.

With liver-normalized SUL thresholds defined, discrimination between tumor and non-tumor uptake is well possible particularly at time-points later than 6 months after irradiation. Tools for this kind of semi-automated evaluation are already available from the well-known software developers and could easily be implemented in every software environment. After being started, the algorithm runs in the background during the visual assessment and especially the workflow of unexperienced readers could benefit from it.

Yet, inflammatory changes within the first 6 months seem to confound threshold-based assessments. In this period, visual assessments show a higher sensitivity. However, it comes at cost of a low specificity, which states that the visual assessment might be conducted too carefully.Even experienced readers tend to be too sensitive and their assessment is overcalling inflammatory changes as recurrence, consecutively resulting in a low specificity and a higher false positive rate. These findings are supported by the low IOA in this time period and line up with the results of studies investigating local therapy response in the lungs after radiotherapy [12, 13]. Investigating local recurrence in the lung within the first 6 months after treatment can be delusive with danger given the low specificity and PET/CT images should always be discussed in clinical context. After 6 months quantitative parameters, when adapted to liver background, show similar sensitivity (86%) and specificity (97%) in diagnosing disease relapse. Visual assessment shows slightly higher sensitivity (92%) and slightly lower specificity (89%) favoring the visual assessment as current gold standard in PET/CT reading for relapse in many situations.

One strength of the study is the well-controlled primary endpoint of the PET-Plan trial, which was time to locoregional progression; secondary endpoints included time to out-of-field progression and time to distant progression amongst others [7]. However, some bias for this retrospective analysis cannot be ruled out, since PET/CT was an integral imaging for verification of the primary endpoint. Moreover, due to the inclusion criteria “suspected relapse on CT”, a bias towards a higher pre-test likelihood of recurrence must be assumed. Conversely, without suspected relapse on CT imaging, patients did not receive follow-up PET/CT imaging in the original study, which could possibly account for a bias in distant recurrence as PET/CT imaging is superior to CT in the detection of distant recurrence. Validation of the results in an independent trial cohort with routine PET/CT imaging following 6 months after therapy would be of further interest.

## Conclusion

In the visual assessment of suspected relapse, interobserver agreement between observers decreases with subjective insecurity. Although additional knowledge of the initial staging PET and radiotherapy delineation volumes improves the sensitivity (0.85 vs 0.92) of the qualitative evaluation slightly, it has no significant impact on the specificity (0.86 vs 0.89). The implementation of a patient individual threshold value definition for the SULpeak as previously discussed by Wahl et al*.* offers a promising approach for a quantitative evaluation in the situation of suspected relapse [19]. Our study supports the visual evaluation of experienced nuclear medicine physicians as the current gold standard in evaluating PET/CT for relapse, especially when it comes to differentiating between therapy effect and recurrence within the first six months after chemoradiotherapy. Nevertheless, thresholds in particular when adapted to individual liver uptake display very similar sensitivities and specificities.

## Data Availability

The datasets generated during and/or analysed during the current study are available from the corresponding author on reasonable request.

## Abbreviations

[18F]FDG PET/CT: fluorine-18-fluorodeoxyglucose positron emission tomography/ computed tomography
CT: computed tomography
ICA: intercriteria agreement
IOA: interobserver agreement
ĸ: kappa
NSCLC: Non-small cell lung cancer
PERCIST: PET Response Criteria in Solid Tumor
PET: positron emission tomography
PREDIST: PET Residual Disease in Solid Tumor
RILD: radiation induced lung disease
ROI: region of interest
SD: standard deviation
SULmean: mean standardized uptake value corrected for lean body mass
SULpeak: peak standardized uptake value corrected for lean body mass
SUV: standardized uptake value
SUVmax: maximum standardized uptake value
